# Draft genome of *Proteus mirabilis* strain UACOMP-J6 isolated from the human endometrium

**DOI:** 10.1128/mra.00464-26

**Published:** 2026-07-20

**Authors:** Nicole R. Jimenez, Melissa M. Herbst-Kralovetz

**Affiliations:** 1 Department of Obstetrics and Gynecology, The University of Arizona College of Medicine-Phoenix 42283, Phoenix, Arizona, USA; 2 Department of Basic Medical Sciences, The University of Arizona College of Medicine-Phoenix 42283, Phoenix, Arizona, USA; Nanchang University, Nanchang, Jiangxi, China

**Keywords:** *Proteus mirabilis*, endometrium, gynecology, urinary tract infection, pathogens, hyperplasia

## Abstract

We sequenced and annotated the genome of *Proteus mirabilis* UACOMP-J6, a human endometrial isolate from a patient with endometrial hyperplasia. UACOMP-J6 had a genome size of 4.1 Mb, GC content of 38.8%, and 3,774 coding sequences. Genome characteristics were highly similar to those of *P. mirabilis* from other niche sites.

## ANNOUNCEMENT

*Proteus mirabilis* is recognized as a pathogen linked to infections, including recurrent UTIs (1–3), Crohn’s disease (4), neonatal septicemia (5), endometritis (6), and fasciitis after hysterectomy (7, 8).

The isolate was obtained from an endometrial swab of a patient with endometrial hyperplasia. Swabs were cultured and isolated on tryptic soy agar with 5% sheep’s blood at 37°C for 48 hours. Pellets were collected from one plate, washed twice by suspension in 1 mL of sterile DPBS, and resuspended in 500 μL of DNA/RNA Shield (Zymo Research, cat. R1100-50). DNA extraction, sequencing, and assembly were performed by Plasmidsaurus, Inc. (9). DNA was extracted according to the Zymo Quick-DNA Miniprep Plus Kit (cat. D4069) protocol. ONT libraries were prepared using the Ligation Sequencing Kit v14 (enzyme E8.2.1) and the DNA was neither sheared nor size-selected. For Illumina, the Illumina DNA Prep Kit (part #20060059) was utilized. Sequencing technologies included Oxford Nanopore Technologies (ONT) R10.4.1 flow cells and paired-end 2 × 150 bp Illumina NovaSeq X Plus. Short reads were processed using fastp (10) v1.3.3 with quality filtering (Phred ≥15, length ≥50 bp, adapter trimming, and poly-G trimming). Basecalling was done using Dorado v4.3 Super-Accurate Basecalling with default Q10 quality filtering (11). Assembly was performed using Autocycler v0.5.2 (12), utilizing Flye v2.9.6 (read error 0.03, genome size 5 Mb) (13), and Hifiasm-0.25.0-r726 for generating high-quality assemblies (14–16). The ONT assembly was polished with Illumina reads using Polypolish v0.6.0 (17, 18). Default parameters for assembly were used except where noted. Taxonomic classification was performed with Sourmash v4.6.1 (19, 20) against Genome Taxonomy Database release 226 (21). The hybrid genome assembly was analyzed with Genome Annotation (RASTtk [22] v2.0), Protein Family Sorter analysis (Patyfams [23]), and Genome Tree (MUSCLE [24] v3.8.31 and RAxML [25] v8.2.11) services of the Bacterial and Viral Bioinformatics Resource Center (26).

SourMash (20) inferred that this sequence was *P. mirabilis*, with an average nucleotide identity of 99.2% to the representative *P. mirabilis* strain HI4320. UACOMP-J6 had an assembled coverage of 357×, a genome size of 4.1 Mb, a GC content of 38.8%, consistent with the representative strain (Table 1), and a plasmid containing 34 hypothetical proteins, seven plasmid proteins, 12 type IV secretion proteins, 2 RelE repressors, and 2 ParE toxin proteins. An additional 47 antimicrobial resistance proteins and 17 virulence factors were observed. UACOMP-J6 functional subsystems revealed that metabolism was the largest subsystem (*n* = 664) (Table 1).

**TABLE 1:** Summary of genome characteristics for *P. mirabilis* UACOMP-J6^*a*^

Genomic information		
Genome name	*P. mirabilis* UACOMP-J6	*P. mirabilis* HI4320 (representative)
Isolate information		
Isolation source	Human endometrium	Human urinary tract
Additional characteristics on source	Endometrial hyperplasia	Tetracycline resistance
Strain identity		
SourMash identity	*Proteus mirabilis*	*Proteus mirabilis*
Average nucleotide identity to representative strain (%)	99.19	100%
Genome characteristics		
Total bp sequenced	1,451,414,265 bp	
Total number of reads	343,616 ONT reads; 27,224,410 Illumina reads	
Assembled coverage	357×	
Raw coverage	105×	
Genome size (bp)	4,061,767	4,099,895
Number of contigs	2	2
Contig N50 (bp)	4,025,738 ONT; 3,966,988 Illumina	4,063,606
Contig L50	1	1
GC (%)	38.76	38.9
Number of 5S rRNA	8	8
Number of 16S rRNA	7	7
Number of 23S rRNA	7	7
Number of tRNAs	87	83
Number of CDS	3,774	3,685
Number of CDS with functional assignments	3,014	2,718
Number of hypothetical proteins	760	967
Plasmids present	1	1
Number of antibiotic resistance genes (PATRIC)	47	57
Number of virulence factor genes (PATRIC)	17	33
Number of unique protein families	5	53
Number of genes related to metabolism (PATRIC)	664	626
Number of genes related to energy (PATRIC)	262	257
Number of genes related to protein processing (PATRIC)	241	236
Number of genes related to stress response, defense, virulence (PATRIC)	157	123
Number of genes related to cellular processes (PATRIC)	138	119
Number of genes related to membrane transport (PATRIC)	123	94
Number of genes related to DNA processing (PATRIC)	75	74
Number of genes related to RNA processing (PATRIC)	74	67
Number of genes related to cell envelope (PATRIC)	52	52
Number of genes related to regulation and cell signaling (PATRIC)	13	13

^*a*^ The genome of *P. mirabilis *UACOMP-J6 was isolated from the endometrium of a patient with endometrial hyperplasia. Genus and species identification were performed by using SourMash and validated by average nucleotide identity. The table summarizes genome assembly statistics, including the number of contigs, total raw reads, N50, genome size, GC content, and genome annotation results. Key genomic features reported include the number of coding sequences, rRNA and tRNA genes, and the proportions of genes across different subsystems. Additionally, using the Bacterial and Viral Bioinformatics Resource Center, we identified the number of unique protein families reported by PATRIC and predicted antibiotic resistance genes, along with their corresponding PATRIC gene identifiers.

**Fig 1:**
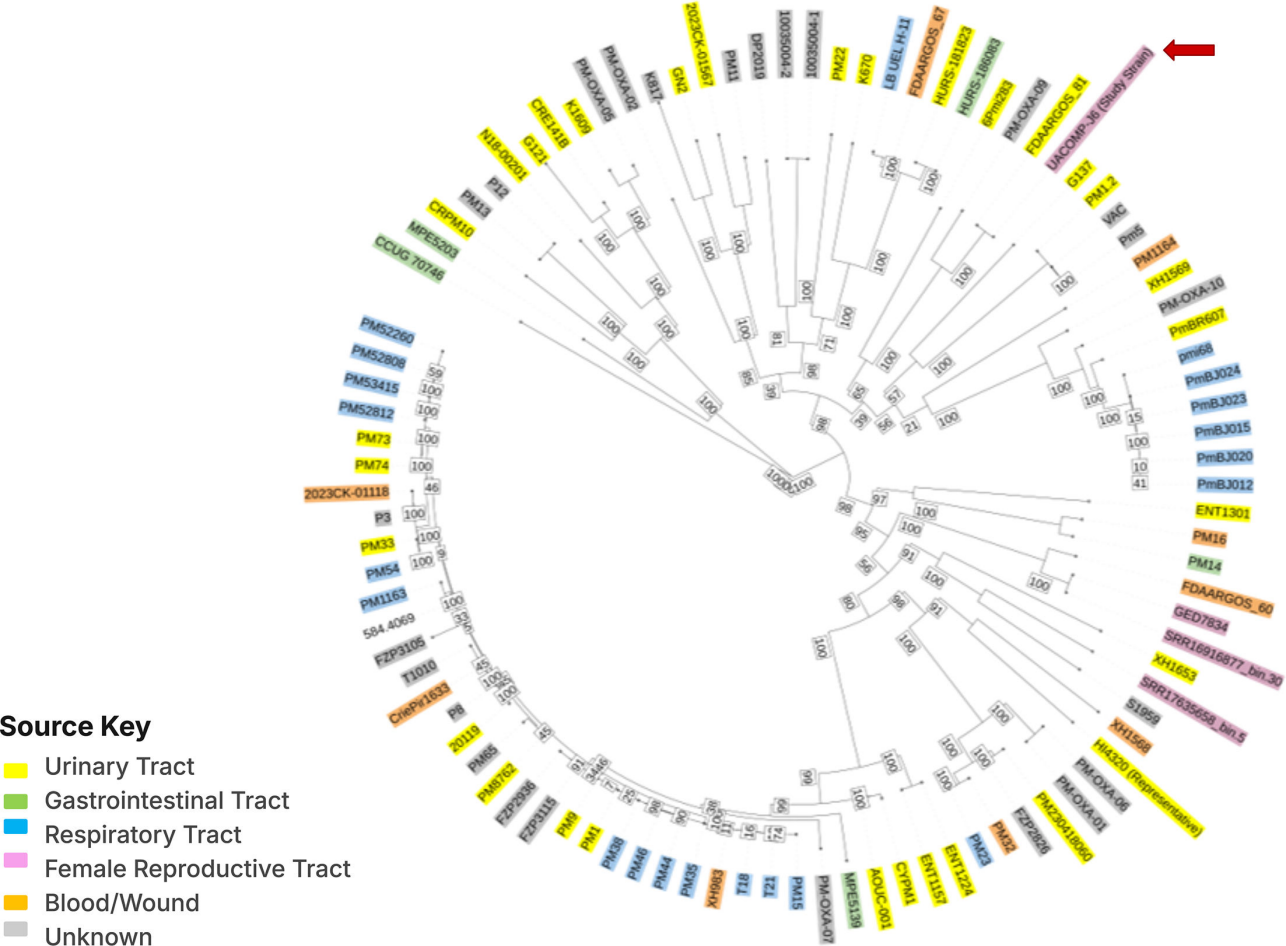
Comprehensive genomic and functional overview of the endometrial *Proteus mirabilis* UACOMP-J6 strain. A circular phylogenetic tree illustrating the relationships of 500 single-copy genes among human and whole-genome *P. mirabilis* isolates (*n* = 93). The outer ring is color-coded by isolation source: urinary tract (*n* = 31) (yellow), gastrointestinal tract (*n* = 5) (green), respiratory tract (*n* = 21) (blue), FRT (*n* = 4) (pink), blood/wound (*n* = 9, orange), and unknown (*n* = 26, gray). These analyses were performed on the Bacterial and Viral Bioinformatics Resource Center, and the phylogenetic tree was visualized using the Interactive Tree of Life.

A phylogenetic tree based on 500 single-copy genes, using all publicly available human-derived whole-genome strains (*n* = 94), revealed no distinct relationships based on isolation source. Because only a limited number of the 93 strains originated from the female reproductive tract, we supplemented the analysis with two metagenome-assembled genomes (*n* = 2) (Fig. 1). UACOMP-J6 is closely related to strains of the urinary tract and of unknown origin. We identified 2,479 protein families shared among strains isolated from humans. Proteins relevant to the FRT included spermidine/putrescine metabolism (*n* = 9), iron acquisition (*n* = 28), anthranilate metabolism (*n* = 4), and aromatic amino acid metabolism (*n* = 19). Unique proteins specific to UACOMP-J6 included four hypothetical ones and a phage-associated protein.

## Data Availability

*P. mirabilis* UACOMP-J6 genome information is also available under BioProject PRJNA1414603 and BioSample SAMN54910878 with raw reads as SRR36991315 (paired end) and SRR36991314 (long read), and PGAP annotations are available at JBUBKS010000001–JBUBKS010000002. The PATRIC annotation and genomic data analysis outputs are publicly available with a free account at the Bacterial and Viral Bioinformatics Resource Center (https://www.bv-brc.org/workspace/public/jimeneznr@patricbrc.org/Proteus_mirabilis_UACOMP-J6).
